# Supplementary material for: Study of the Variation of Phenolic Acid and Flavonoid Content from Fresh Artemisiae argyi Folium to Moxa Wool
Source: Molecules. 2019 Dec 16;24(24):4603. doi: 10.3390/molecules24244603 (PMC6943600; doi:10.3390/molecules24244603)
Supplement: Supplementary file 1 [file molecules-24-04603-s001.pdf]

Supplementary Materials to:

Study of the Variation of Phenolic Acids and Flavonoids Content from Fresh *Artemisiae argyi Folium* to Moxa Wool

Min Li, Xin Chai, Luyao Wang, Jing Yang*, Yuefei Wang*

Tianjin Key Laboratory of TCM Chemistry and Analysis, Tianjin University of Traditional Chinese Medicine, Tianjin 301617, China; lm7911826@163.com (M.L.); chaix0622@tjutcm.edu.cn (X.C.); W1224967197@163.com (L.W.); yangj0622@tjutcm.edu.cn (J.Y.); wangyf0622@tjutcm.edu.cn (Y.W.)

* Corresponding author. Tianjin Key Laboratory of TCM Chemistry and Analysis, Tianjin University of Traditional Chinese Medicine, No.10 Poyanghu Road, Jinghai district, Tianjin 301617, China. yangj0622@tjutcm.edu.cn (J.Y.); wangyf0622@tjutcm.edu.cn (Y.W.); Tel.: +86-22-59596366

Table S1. The detailed information of AAF collected from the different habitats

Batch No.	Habitat
HN-1	Henan, China
HN-2	Henan, China
HN-3	Henan, China
HN-4	Henan, China
HN-5	Henan, China
HN-6	Henan, China
HN-7	Henan, China
HN-1	Henan, China
HN-2	Henan, China
HN-3	Henan, China
HQ-1	Qichun (Hubei, China)
HQ-2	Qichun (Hubei, China)
LN-1	Liaoning, China
LN-2	Liaoning, China
HB-1	Hebei, China
HB-2	Hebei, China

Table S2. The detailed information of moxa wool

Batch No.	AAF/Moxa wool	Origin
HC 1-1	1:1	Hebei, China
HC 1-2	1:1	Hebei, China
HC 1-3	1:1	Hebei, China
HC 2-1	5:1	Hebei, China
HC 2-2	5:1	Hebei, China
HC 2-3	5:1	Hebei, China
HC 3-1	10:1	Hebei, China
HC 3-2	10:1	Hebei, China
HC 3-3	10:1	Hebei, China
HC 4-1	15:1	Hebei, China
HC 4-2	15:1	Hebei, China
HC 4-3	15:1	Hebei, China
